# Investigating the interpretability of fetal status assessment using antepartum cardiotocographic records

**DOI:** 10.1186/s12911-021-01714-4

**Published:** 2021-12-20

**Authors:** Liting Huang, Zhiying Jiang, Ruichu Cai, Li Li, Qinqun Chen, Jiaming Hong, Zhifeng Hao, Hang Wei

**Affiliations:** 1 School of Computer, Guangdong University of Technology, Waihuan West Road, Guangzhou, China; 2 School of Medical Information Engineering, Guangzhou University of Chinese Medicine, Waihuandong Road, Guangzhou, China; 3 The First Affiliated Hospital of Jinan University, Tianhe District People’s Hospital, Dongpu Road, Guangzhou, China; 4 Department of Mathematics, College of Science, Shantou University, Daxue Road, Shantou, 515063 China; 5 Guangzhou Sanrui Medical Equipment Co, Gaoke Road, Guangzhou, China

**Keywords:** Fetal monitoring, Cardiotocography, Forward-stepwise-selection association rule analysis, Structural equation model

## Abstract

**Background:**

Cardiotocography (CTG) interpretation plays a critical role in prenatal fetal monitoring. However, the interpretation of fetal status assessment using CTG is mainly confined to clinical research. To the best of our knowledge, there is no study on data analysis of CTG records to explore the causal relationships between the important CTG features and fetal status evaluation.

**Methods:**

For analyses, 2126 cardiotocograms were automatically processed and the respective diagnostic features measured by the Sisporto program. In this paper, we aim to explore the causal relationships between the important CTG features and fetal status evaluation. First, we utilized data visualization and Spearman correlation analysis to explore the relationship among CTG features and their importance on fetal status assessment. Second, we proposed a forward-stepwise-selection association rule analysis (ARA) to supplement the fetal status assessment rules based on sparse pathological cases. Third, we established structural equation models (SEMs) to investigate the latent causal factors and their causal coefficients to fetal status assessment.

**Results:**

Data visualization and the Spearman correlation analysis found that thirteen CTG features were relevant to the fetal state evaluation. The forward-stepwise-selection ARA further validated and complemented the CTG interpretation rules in the fetal monitoring guidelines. The measurement models validated the five latent variables, which were baseline category (BCat), variability category (VCat), acceleration category (ACat), deceleration category (DCat) and uterine contraction category (UCat) based on fetal monitoring knowledge and the above analyses. Furthermore, the interpretable models discovered the cause factors of fetal status assessment and their causal coefficients to fetal status assessment. For instance, VCat could predict BCat, and UCat could predict DCat as well. ACat, BCat and DCat directly affected fetal status assessment, where ACat was the important causal factor.

**Conclusions:**

The analyses revealed the interpretation rules and discovered the causal factors and their causal coefficients for fetal status assessment. Moreover, the results are consistent with the computerized fetal monitoring and clinical knowledge. Our approaches are conducive to evidence-based medical research and realizing intelligent fetal monitoring.

## Background

Cardiotocography (CTG) was widely introduced into antenatal fetal monitoring in the late 1960s and is still widely used due to its low cost, ease of operation and non-invasiveness [1]. Through recording fetal heart rate (FHR) and uterine contraction (UC) signals in non-stress testing (NST) [2, 3], it helps early diagnosis of pathological cases, such as congenital heart defects, fetal distress and hypoxia, which can timely prevent irreversible damage to the fetus [4].

The mainstream fetal monitoring guidelines include Society of Obstetricians and Gynaecologists of Canada (SOGC) [5], American College of Obstetricians and Gynecologists (ACOG), National Institute for Health and Care Excellence (NICE), International Federation of Obstetrics and Gynecology (FIGO) [6, 7], and Chinese expert consensus [8]. Obstetricians usually assess fetal health status by visually interpreting the morphological CTG features, including baseline, variation, deceleration and so on, and then apply them to the corresponding fetal monitoring guidelines. However, visual analysis of CTG records by guidelines usually leads to high intra-observer and inter-observer disagreement among obstetricians [9]. Thus some researchers focused on developing the automated CTG analysis software to extract CTG features from FHR and UC signals. For instance, SYSTEM 8000 was developed for antenatal FHR analysis [10]. Open-access software CTG-OAS was introduced for the automatic analysis of FHR signals [11]. CTG Analyzer was proposed as a graphical tool for automatic and objective analysis of CTG tracings [12]. The Sisporto program was developed to analyze FHR and UC signals automatically and used a relatively complex algorithm to estimate the mean FHR during periods of fetal rest without reducing signals, which can evaluate the closest variability of beat-to-beat [13, 14].

With the computerized CTG analysis technology, some researchers combined CTG features with clinical theoretical studies. They identified biomarkers on CTG records that helped to diagnosis of pathological cases, like distress, growth restriction and cardiac arrhythmia. For example, fetal distress was considered to be associated with variable deceleration and hyperdynamic uteri [15]. Meanwhile, some maternal diseases could be judged by prenatal CTG features [4].

In recent decades, many researchers applied machine learning methods to classify CTG data for fetal status assessment. The study [16] evaluated the classification performance of eight different machine learning methods on prenatal CTG data, which showed the Bagging with Random Forest achieved better results with an accuracy of 99.02%. Yang et al. used hybrid principal component analysis (PCA) and adaptive enhancement (AdaBoost) to successfully classify CTG data and assessed fetal status with an accuracy of 98.6% [17]. In literature [18], Vinayaka Nagendra used RF and support vector machine (SVM) to perform a three-categories study of the fetal status, and the results showed that the accuracy was higher than 96% . Zhao et al. used statistical test (ST), the area under the curve (AUC) and PCA to select features, and applied three representative machine learning algorithms, decision tree (DT), the SVM and AdaBoost to classify the fetal status as two categories [19].

The above studies focused on the relationships between CTG features and fetal status or diseases in clinical research, or directly established evaluation models by machine learning methods. However, they did not pay attention to the interpretability of fetal status assessment. Consequently, it is significant to investigate the interpretability of fetal status assessment using cardiotocographic records. In this study, data visualization, Spearman correlation analysis, forward-stepwise-selection ARA, and SEM models were implemented to achieve the following goals: (1) identifying important CTG features of great impact on fetal status; (2) exploring the association rules between CTG features and fetal status to supplement fetal monitoring guidelines; (3) exploring the causal relationship among latent variables and the causal relationship of latent variables to fetal status.

## Methods

### Data and analysis tools

**Fig. 1:**
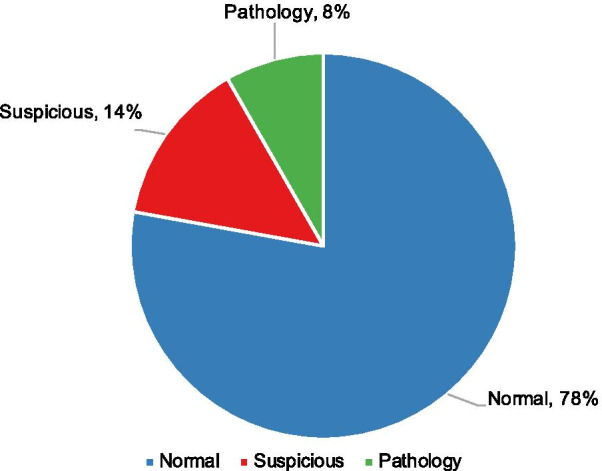
The prenatal CTG data distribution. The fetal status: normal, suspicious and pathology cases account for 78%, 14% and 8%, respectively

For analyses, we selected the cardiotocography (CTG) dataset from the public available UCI Machine Learning Repository [20], which has been validated by numerous machine learning-based fetal state assessment researches with the accuracy of higher than 95% [16–19]. In the dataset, 2126 CTG signals with 29–42 gestational weeks were automatically processed and the respective 21 diagnostic features were measured by the SisPorto2.0 program undergoing a multicenter validation study (http://sisporto.med.up.pt) [13]. The FHR feature computation was based on consensual guidelines, such as the International Federation of Gynecology and Obstetrics (FIGO) and the National Institutes of Health. It involved 11 steps in successive order, including FHR spike removal, filtering of uterine contraction signals, detection of uterine contractions, and so on. The attribute information of 21 diagnostic features can be found in Table 1. The CTGs were also interpreted by three expert obstetricians according to the FIGO guideline shown in Table 2 and a consensus classification label concerning fetal state (NSP: normal, suspicious and pathology) assigned to each of them. In all, there are 1655, 295 and 176 instances for each category respectively. Figure 1 shows that the proportion of the categories was imbalanced. The normal, suspicious and pathology cases accounted for 78%, 14% and 8%, respectively.

Data visualization, Spearman correlation analysis and forward-stepwise-selection ARA were implemented using programming language Python3.8 and the compilers Pycharm Professional 2020.3.2. The measurement model and the structural equation model were analyzed by AMOS 24.0 software.

**Table 1:** Attribute Information about the CTG dataset

Symbol	Feature description
LB	FHR baseline (beats per minute)
AC	Number of accelerations per second
FM	Number of fetal movements per second
UC	Number of uterine contractions per second
DL	Number of light decelerations per second
DS	Number of severe decelerations per second
DP	Number of prolonged decelerations per second
ASTV	Percentage of time with abnormal short term variability
MSTV	Mean value of short term variability
ALTV	Percentage of time with abnormal long term variability
MLTV	Mean value of long term variability
Width	Width of FHR histogram
Min	Minimum of FHR histogram
Max	Maximum of FHR histogram
Nmax	Number of histogram peaks
Nzeros	Number of histogram zeros
Mode	Histogram mode
Mean	Histogram mean
Median	Histogram median
Variance	Histogram variance
Tendency	Histogram tendency
NSP	Fetal state class code (N = 1; S = 2; P = 3)

**Table 2:** The definition of three categories of fetal status (normal, suspicious and pathology) according to the FIGO guidelines

	Normal	Suspicious	Pathology
Baseline	Between 110 and 150 bpm	Between 100 and 110 bpm or between 150 and 170 bpm	Below 100 or above 170 bpm
Variablity	Between 5 and 25 bpm	Amplitude of variability between 5 and 10 bpm for more than 40 s	Persistence of heart rate variability of less than 5 bpm for more than 40 s
Others		Increased variability above 25 bpm; Variable decelerations	Severe variable decelerations or severe repetitive early decelerations; Prolonged decelerations; Late decelerations: the most ominous trace is a steady baseline without baseline variability and with small decelerations after each contraction; A sinusoidal pattern

### Correlation and significance analysis

The relationships among CTG features and those between CTG features and fetal status (NSP) were discussed by Spearman’s rank correlation coefficient, which measured the degree of correlation between grading sequencing variables.

### Forward-stepwise-selection ARA

Normally, pathology cases are seriously scarce in clinical practice. While the traditional Apriori algorithms [21, 22] in ARA can not achieve the infrequent association rules and the improved method based on increasing the weight of minority would produce biased results as well [6, 23]. Therefore we designed an algorithm of forward-stepwise-selection association rule to maximize the accuracy of association rules. The CTG features were firstly discretized by fetal status distribution and the basic flow was shown in Algorithm 1.
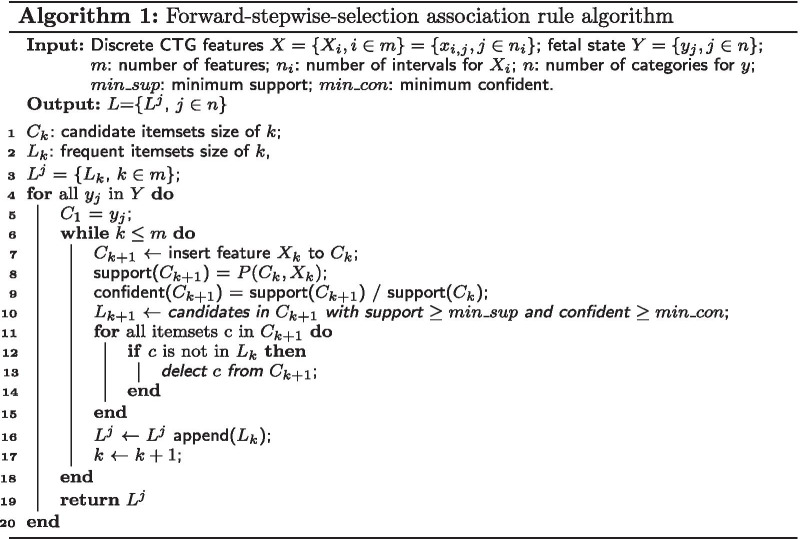


### SEMs for interpreting prenatal fetal monitoring

**Fig. 2:**
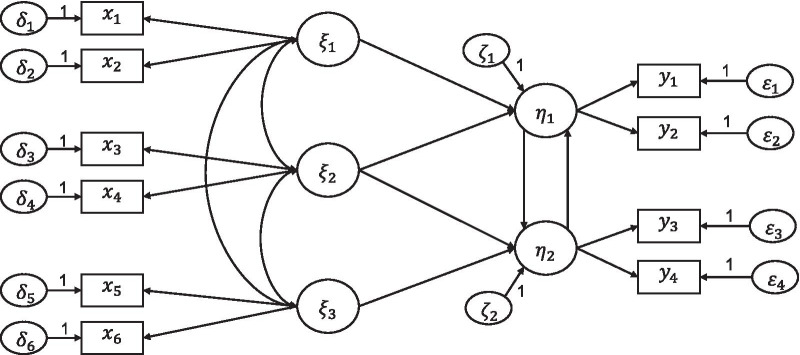
An hypothetical example of a structural equation model. The single-headed arrow represents causal relationship, the double-headed arrow indicates unresolved causality

Structural equation modeling is referred to as covariance structural analysis that combines confirmatory factor analysis (CFA) and regression or path analysis. First, the latent variables that can be represented by their observed variables were assessed by estimating the measurement models. If the measurement models satisfied the fitting indices requirements, the final measurement model would be adopted to investigate the impact of latent variables. Furthermore, structural equation models (SEMs) were introduced to study the relationship between the observed variables and the latent variables, the causation among latent variables, and the mechanism of the influence of each latent variable on fetal status. The theoretical model of SEMs is shown in Fig. 2. The relationships between observation variables and latent variables are expressed as Eqs. (1) and (2).

1 $$\begin{aligned} x&=\varvec{\Lambda _x}\xi + \varvec{\delta } \end{aligned}$$

2 $$\begin{aligned} y&=\varvec{\Lambda _y}\eta + \varvec{\epsilon } \end{aligned}$$

where $x$ and $y$ are prenatal fetal monitoring features, $\xi$ and $\eta$ are latent variables, $\delta$ and $\epsilon$ are unique factor vectors. The simple form of the structural equation model is a multiple regression model with only one dependent variable. The Eq. (3) is as follows, where $\eta$ is determined by y and $\epsilon$.

3 $$\begin{aligned}&\eta =\varvec{\beta }\eta + \varvec{\Gamma }\xi +\zeta \end{aligned}$$

## Results

### Exploration of the important CTG features

**Fig. 3:**
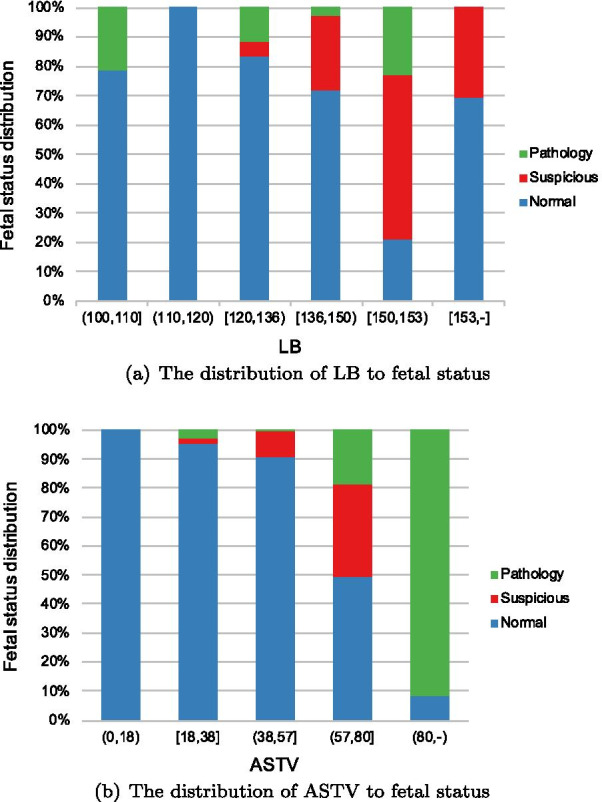
Distribution of fetal status to features proportion

The proportion of the fetal status distribution within a certain range for each CTG feature was visualized by percentage stacked bar charts. The experimental data visualization revealed the following findings. When the values of AC, UC and MSTV increased, the fetal status tended to be normal. In contrast, when the values of ASTV, ALTV, DP and LB increased, the fetus was more likely to be pathological. Figure 3 illustrates two instances. Firstly, the baseline (LB) values in the normal cases were mostly at the interval of 111–120 bpm, while the numbers of suspicious and pathology cases increased with baseline values. Secondly, the proportion of pathology cases increased with ASTV, whereas that of normal reversed; when ASTV value ranged from 0 to 18, the proportion of normal category reached 100%; as the ASTV value exceeded 80, the proportion of pathological cases increased significantly. Overall, twelve CTG features symbolized as AC, UC, LB, FM, DP, ASTV, ALTV, MLTV, Mode, Mean, Min, and Median were closely related to the fetal status.

**Fig. 4:**
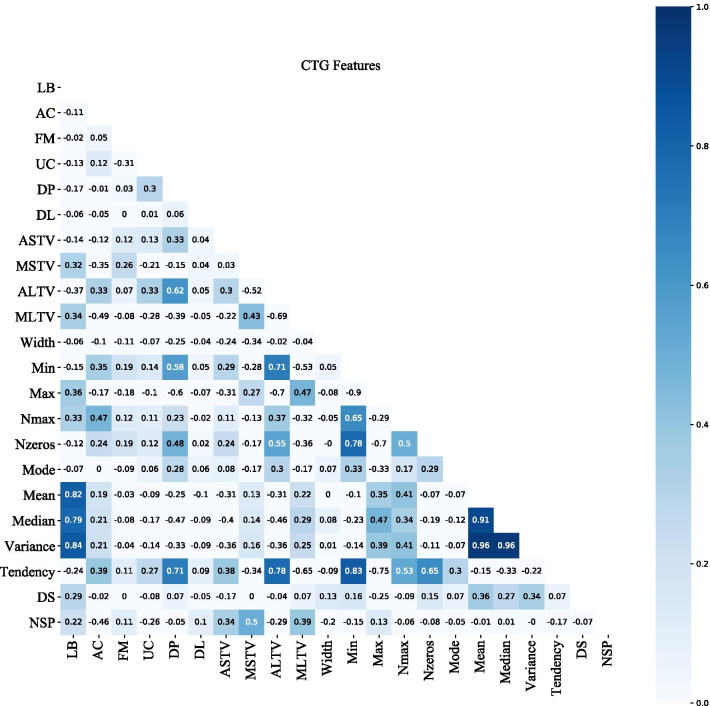
The correlation coefficient matrix of CTG features and fetal status

Figure 4 is a thermal map calculated by Spearman’s coefficient, showing highly correlated feature groups and important features associated with fetal status. Ten features with high correlation among the 21 features were divided into two groups listed in Table 3. In addition, the CTG features correlated with pathological fetal were found to be MSTV, MLTV and ASTV ($R = 0.5$, 0.39 and 0.34, respectively); the features associated with normal fetal were AC, ALTV and UC ($R = -0.46, -0.29$ and $-0.26$, respectively).

**Table 3:** Grouping of ten highly correlated CTG features

Group	Correlated feature
1	LB, Mode, Median, Mean
2	Width,Variance, Nmax, MSTV, Max, DL

### Supplement of the fetal monitoring guidelines

We explored the relationship between CTG features and fetal status using forward-stepwise-selection association rules. The results showed that MSTV, AC, Mean, ASTV and Min were the decisive features to normal fetal status. When the values of those were within certain ranges, the confidence values for normal fetal state attained 100%. On the other hand, the key features to pathological fetal status were DP, DS, Variance, and Mean. When the values of those ranged in certain intervals, the confidence values of fetal status for pathology reached 100%. In summary, the CTG interpretation rules excavated by the proposed ARA verified and supplemented the fetal monitoring guidelines displayed in Table 4.

**Table 4:** Supplement to the fetal monitoring guidelines

Fetal status	FIGO	Supplement
Normal	MSTV: Average STV^a^; ASTV: percentage of points with STV	AC: [0.0048, 0.005] times per second; MSTV: $\left[ 0.6, 1.5\right)$; Mean: $\left[ 125.175, 138.23\right)$; Min: $\left[ 86, 108\right)$; ASTV: $\left[ 30, 44\right)$
Pathology	DS: lasting at least 15 s and with amplitude exceeding 15 bpm; DP: lasting 120–300 s with amplitude exceeding 15 bpm	Mode: range in [60, 99); Variance: $>165$; DS: $> 0.00094$ times per second; DP: $>0.0028$ times per second

^a^STV is identified whenever the difference between two adjacent FHR signals is less than 1 bpm

### The explanation mechanism of prenatal CTG feature on fetal status

#### The result of measurement model assumptions

**Fig. 5:**
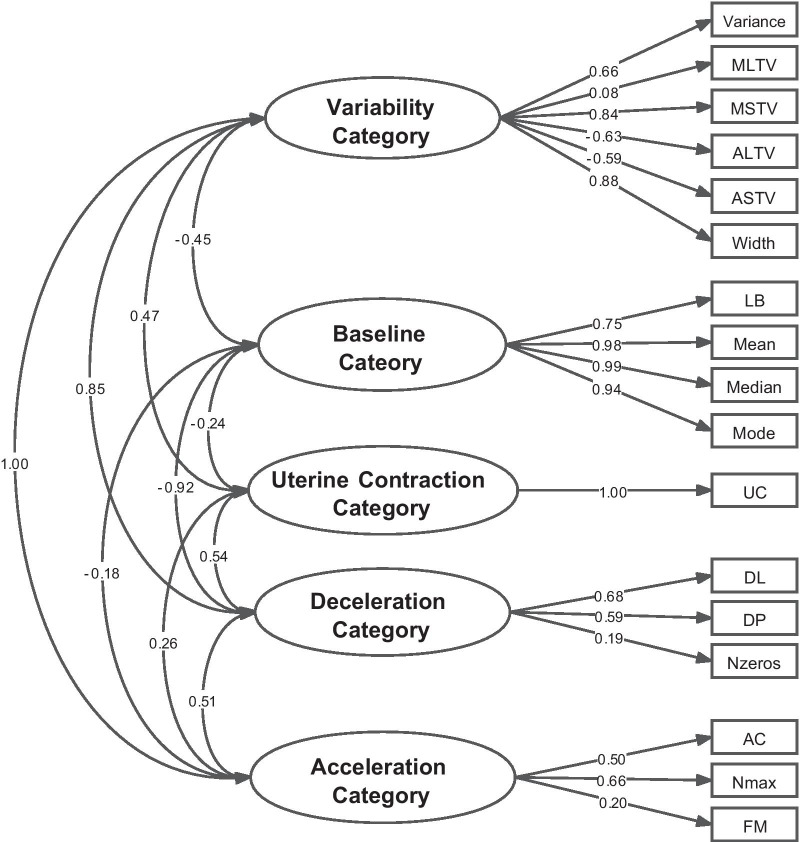
The measurement model for five latent constructs and seventeen observed variables. Note: The values on an arrow are standard path coefficients (also known as regression coefficients); the values on double arrows indicate the correlationships among the five latent variables

Combined with FIGO guidelines and clinical knowledge [24, 25], seventeen features with great influence on fetal status were obtained through data visualization, Spearman correlation analysis and forward-stepwise-selection association rules. On this basis, seventeen features were divided into five categories named baseline category (BCat), variability category (VCat), acceleration category (ACat), deceleration category (DCat) and uterine contraction category (UCat). And a measurement model (Fig. 5) assuming all features could be explained by their latent variables (categories) was established. The measurement model using the oblique confirmatory factor analysis method not only verified whether the latent variables we hypothesized had meaningful interpretation to the observed variables, but also explored the correlation among the five latent variables in this study. In Table 5, except for the MLTV, the other 16 features were all statistically significant ($P<0.001$), confirming our hypotheses that these five latent variables were meaningful in explaining their respective observed variables [26].

**Table 5:** Standardized regression weighting coefficients and parameter estimates for observed variables to latent variables

Hyoithesized path	Estimate^a^	C.R.^b^	*P* values^c^
Variance $\longleftarrow$ VCat	0.662		
MLTV $\longleftarrow$ VCat	0.084	2.296	0.282
MSTV $\longleftarrow$ VCat	0.837	20.662	***
ALTV $\longleftarrow$ VCat	− 0.632	− 16.244	***
ASTV $\longleftarrow$ VCat	− 0.589	− 15.248	***
Width $\longleftarrow$ VCat	0.880	21.493	***
LB $\longleftarrow$ BCat	0.748		
Mean $\longleftarrow$ BCat	0.977	30.763	***
Median $\longleftarrow$ BCat	0.990	31.232	***
Mode $\longleftarrow$ BCat	0.937	29.210	***
DL $\longleftarrow$ DCat	0.680	16.818	***
DP $\longleftarrow$ DCat	0.586		
Nzeros $\longleftarrow$ DCat	0.191	5.561	***
AC $\longleftarrow$ ACat	0.497		
Nmax $\longleftarrow$ ACat	0.660	14.527	***
FM $\longleftarrow$ ACat	0.201	5.891	***
UC $\longleftarrow$ UCat	1.000		
VCat $\longleftrightarrow$ BCat	− 0.447	− 9.549	***
DCat $\longleftrightarrow$ ACat	0.51	7.004	***
VCat $\longleftrightarrow$ DCat	0.848	11.498	***
VCat $\longleftrightarrow$ ACat	1.00	11.556	***
BCat $\longleftrightarrow$ DCat	− 0.921	− 12.737	***
BCat $\longleftrightarrow$ ACat	− 0.178	− 3.547	***
UCat $\longleftrightarrow$ BCat	− 0.241	− 6.438	***
UCat $\longleftrightarrow$ DCat	0.541	10.042	***
UCat $\longleftrightarrow$ ACat	0.26	5.113	***
UCat $\longleftrightarrow$ VCat	0.467	10.366	***

***Significant at $$P<0.001$$

^a^Standardized regression coefficients of estimate

^b^Critical ratio (C.R.) is the *t* value of T-test

^c^$P>0.05$ Implied that the hypothetical pathways were false

#### Interpretability of prenatal fetal status assessment

**Fig. 6:**
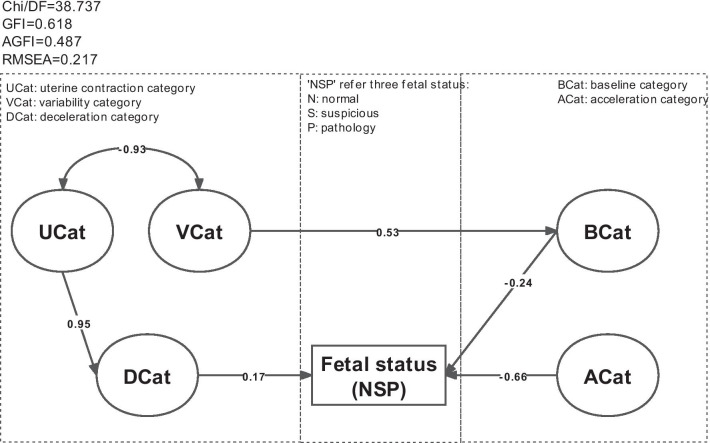
The structural model for analyzing main factors affecting fetal status evaluation and their path ways. Note: The value of standard path coefficient is indicated by an arrow on one end along with a number; the value of correlation coefficient between Vcat and Ucat is represented by double arrow with a number

The purpose of this part was to derive the mechanism of prenatal CTG features and fetal status. Structural equation modeling was proved to be an effective tool to recognize the different impacts of five latent variables on fetal status evaluation. After the model fitting adjustment [27, 28], the structure model was obtained as shown in Fig. 6.

Table 6 lists the results of the structural equation model. All the hypothesis relationships, including the interpretation of latent variables on observed variables, the causality among latent variables, and the influence of latent variables on fetal status were statistically significant ($P<0.05$) [29, 30]. Table 6 and Fig. 6 show that ACat, BCat, and DCat had direct effects on fetal status with the standardized path coefficients of $-\,0.665, -\,0.240$, and 0.173 ($P<0.05$), respectively. It indicated that the fetal status changes from normal to pathology as DCat increases, whereas it has opposite change as ACat and BCat increase. In addition, UCat and VCat were high correlated ($r = -\,0.925$ ) and had an indirect impact on the fetal status by affecting DCat and BCat with the standardized indirect effect of 0.95 and 0.53, respectively.

**Table 6:** Standardized path coefficients and *P* values of the final structural equation model

Relationship	Estimate^a^	C.R.^b^	*P* Values^c^
Variance $\longleftarrow$ VCat	− 0.700	− 15.527	***
MSTV $\longleftarrow$ VCat	− 0.886	− 18.369	***
ALTV $\longleftarrow$ VCat	0.608		
ASTV $\longleftarrow$ VCat	0.531	13.032	***
Width $\longleftarrow$ VCat	− 0.805	− 17.849	***
LB $\longleftarrow$ BCat	0.754		
Mean $\longleftarrow$ BCat	0.975	30.976	***
Median $\longleftarrow$ BCat	0.992	31.908	***
Mode $\longleftarrow$ BCat	0.936	29.481	***
DP $\longleftarrow$ DCat	0.535		
DL $\longleftarrow$ DCat	0.755	13.012	***
Nzeros $\longleftarrow$ DCat	0.345	7.690	***
AC $\longleftarrow$ ACat	0.734		
Nmax $\longleftarrow$ ACat	0.427	7.309	***
FM $\longleftarrow$ ACat	0.166	3.790	***
UC $\longleftarrow$ UCat	0.285		
BCat $\longleftarrow$ VCat	0.527	11.532	***
DCat $\longleftarrow$ UCat	0.952	10.599	***
UCat $\longleftrightarrow$ VCat	− 0.925	− 10.411	***
NSP $\longleftarrow$ ACat	− 0.665	− 7.718	***
NSP $\longleftarrow$ BCat	− 0.240	− 5.423	***
NSP $\longleftarrow$ DCat	0.173	2.856	0.004

***Significant at $$P<0.001$$

^a^Standardized path coefficients of estimate

^b^Critical ratio (C.R.) is the *t* value of T-test

^c^$P>0.05$ implied that the hypothetical pathways were false

## Discussion

Data visualization and Spearman correlation analysis found some important features to fetal state. Visual analysis was conducted from the perspective of data distribution and obtained twelve important features affecting fetal status: AC, UC, LB, FM, DP, ASTV, ALTV, MLTV, Mode, Mean, Min, and Median. It was also found that features related to pathological fetal were MSTV, MLTV, and ASTV, and features related to normal fetal state were AC, ALTV and UC. Combining the results of data visualization and Spearman correlation analysis, the CTG features associated with fetal status are listed as follows: MSTV, AC, MLTV, ASTV, ALTV, UC, LB, Min, FM, DP, Mode, Mean, and Median. In detail, visual analysis and Spearman’s correlation analysis found that as the value of heart rate acceleration (AC) increased, the probability of normal state increased, whereas that of suspicious and pathological state reversed. It can be learned that the heart rate acceleration is an important factor to reflect fetal state and accompanies fetal movement to a certain extent. In addition, data visualization and Spearman correlation analysis found that as the percentage of time with abnormal short-term variability (ASTV) increased, the probability of pathology increased and that of the normal gradually decreased.

Forward-stepwise-selection ARA discovered eight essential features and two discriminant rules about assessing normal and pathological fetal status, which verified and complemented the CTG interpretation rules in fetal monitoring guidelines. The results imply that MSTV, AC, Mean, ASTV and Min are the decisive features of normal fetal status, and the main features of pathological fetal status are DP, DS, Variance, and Mean. From data mining perspective, the outcomes of forward-stepwise-selection ARA further validate what the proposed data visualization and Spearman correlation analysis found.

Combining the above analysis with clinical knowledge of fetal monitoring, we obtain 17 features to establish measurement models and structural equation models for analyzing the interpretable mechanisms of evaluating fetal status. The results of measurement models showed that the significant features of 16 passed the hypothesis test and were grouped into five latent variables, namely ACat, BCat, VCat, DCat and UCat. Consequently, from the outcomes of the structural equation model, it can be observed that ACat directly affects fetal status with the standardized path coefficient $-\,0.665$, which reveals that the fetal status is more likely to be identified as normal with the value of ACat increases. In addition, VCat and UCat influence fetal status by affecting BCat and DCat, respectively.

In summary, our study obtained important CTG features for interpreting fetal status and the mechanistic pathways. It reveals that heart rate acceleration and ASTV are key factors that affect fetal status. The international medical guidelines report that the normal state should satisfy that acceleration increases at least 15 seconds when the baseline is higher than 15 bpm and continues at least twice in 15 minutes [31, 32]. Street et al. found that STV was strongly associated with metabolic acidosis and dead fetus in the uterus when without high variability [33]. In addition, BCat and DCat are mediating factors that affect fetal status through VCat and UCat, respectively. These findings were verified by the clinical knowledge of prenatal fetal monitoring: Uterine contraction blocks the blood flow between the uterus placenta and the decrease of fetal oxygen supply leads to a slow fetal heart rate [34]; Baseline was adjusted by the VCat in SisPorto [13]. These causal relationships between the latent variables and fetal status are beneficial to evidence-based medical research and provide an interpretable reference for fetal status assessment in prenatal fetal monitoring.

## Conclusion

This study analyzed the important CTG features related to the fetal status, explored association rules to supplement fetal monitoring guidelines and causal relationships among latent variables to fetal status using CTG records in different perspectives. Visual analysis was carried out from the perspective of data distribution. Spearman correlation analysis identified important CTG features of great impacts on fetal status. Forward-stepwise-selection ARA excavated the important features and interpretation rules to judge the fetal status as normal and pathological, which verified and supplemented the fetal monitoring guidelines. Structural equation model was used to explore the mechanism of prenatal CTG feature on fetal status, providing an interpretable reference for fetal status assessment in prenatal fetal monitoring.

However, the findings in this study should be interpreted with caution. Firstly, there are various methods for CTG feature computing and analysis, like time-domain and frequency-domain analysis of CTG and FHR recordings. Besides, the same feature or parameter could be defiantly measured by different algorithmic instruments [7, 35]. Hence, the analysis of CTG records needs to be integrated with other clinical information for a comprehensive interpretation and adequate management. In our study, time-domain morphological features of fetal heart rate and contraction signals measured by the Sisporto program were analyzed. Lastly, except for fetal status assessment, one of the main goals in cardiotocography is the investigation of the linkage between antepartum CTG features and postpartum clinical outcomes [36, 37]. Therefore, our further study will design prospective trials to track the clinical fetal outcome for exploring the relationships between the CTG features and some specific fetal diseases, such as fetal distress, intrauterine growth restriction or neonatal academia.

## Data Availability

The data used in this work is publicly available from https://archive.ics.uci.edu/ml/datasets/cardiotocography.

## Abbreviations

AC: Number of accelerations per second
ACat: Acceleration category
ACOG: American College of Obstetricians and Gynecologists
AdaBoost: Adaptive enhancement
ALTV: Percentage of time with abnormal long term variability
ARA: Association rule analysis
ASTV: Percentage of time with abnormal short term variability
AUC: Area under the curve
BCat: Baseline category
CTG: Cardiotocography
DCat: Deceleration category
DL: Number of light decelerations per second
DP: Number of prolonged decelerations per second
DS: Number of severe decelerations per second
DT: Decision tree
FHR: Fetal heart rate
FIGO: International Federation of Gynaecology and Obstetrics
FM: Number of fetal movements per second
LB: FHR baseline (beats per minute)
Max: Maximum of FHR histogram
Mean: Histogram mean
Median: Histogram median
Min: Minimum of FHR histogram
MLTV: Mean value of long term variability
Mode: Histogram mode
NICE: National Institute for Health and Care Excellence
Nmax: Number of histogram peaks
NSP: Normal, suspicious and pathology cases
NST: Non-stress testing
Nzeros: Number of histogram zeros
PCA: Principal component analysis
SEMs: Structural equation models
SOGC: Society of Obstetricians and Gynaecologists of Canada
SVM: Support vector machine
ST: Statistical test
Tendency: Histogram tendency
UC: Number of uterine contractions per second
UCat: Uterine contraction category
Variance: Histogram variance
VCat: Variability category
Width: Width of FHR histogram
